# Inter-observer variability in mammography screening and effect of type and number of readers on screening outcome

**DOI:** 10.1038/sj.bjc.6604954

**Published:** 2009-03-03

**Authors:** L E M Duijm, M W J Louwman, J H Groenewoud, L V van de Poll-Franse, J Fracheboud, J W Coebergh

**Affiliations:** 1 Department of Radiology, Catharina Hospital, PO Box 1350, Eindhoven 5602 ZA, The Netherlands; 2 Comprehensive Cancer Centre South (IKZ)/Eindhoven Cancer Registry, PO Box 231, Eindhoven 5600 AE, The Netherlands; 3 Department of Public Health, Erasmus MC University Medical Center, PO Box 2040, Rotterdam 3000 CA, The Netherlands; 4 Centre of Expertise Transitions in Care, University of Applied Sciences, PO Box 25035, Rotterdam 3001 HA, The Netherlands; 5 Centre of Research on Psychology in Somatic Diseases (CoRPS), Tilburg University, PO Box 90153, Tilburg 5000 LE, The Netherlands

**Keywords:** mammography, screening, radiologist, radiographer, inter-observer

## Abstract

We prospectively determined the variability in radiologists' interpretation of screening mammograms and assessed the influence of type and number of readers on screening outcome. Twenty-one screening mammography radiographers and eight screening radiologists participated. A total of 106 093 screening mammograms were double-read by two radiographers and, in turn, by two radiologists. Initially, radiologists were blinded to the referral opinion of the radiographers. A woman was referred if she was considered positive at radiologist double-reading with consensus interpretation or referred after radiologist review of positive cases at radiographer double-reading. During 2-year follow-up, clinical data, breast imaging reports, biopsy results and breast surgery reports were collected of all women with a positive screening result from any reader. Single radiologist reading (I) resulted in a mean cancer detection rate of 4.64 per 1000 screens (95% confidence intervals (CI)=4.23–5.05) with individual variations from 3.44 (95% CI=2.30–4.58) to 5.04 (95% CI=3.81–6.27), and a sensitivity of 63.9% (95% CI=60.5–67.3), ranging from 51.5% (95% CI=39.6–63.3) to 75.0% (95% CI=65.3–84.7). Sensitivity at non-blinded, radiologist double-reading (II), radiologist double-reading followed by radiologist review of positive cases at radiographer double-reading (III), triple reading by one radiologist and two radiographers with referral of all positive readings (IV) and quadruple reading by two radiologists and two radiographers with referral of all positive readings (V) were as follows: 68.6% (95% CI=65.3–71.9) (II); 73.2% (95% CI=70.1–76.4) (III); 75.2% (95% CI=72.1–78.2) (IV), and 76.9% (95% CI=73.9–79.9) (V). We conclude that screener performance significantly varied at single-reading. Double-reading increased sensitivity by a relative 7.3%. When there is a shortage of screening radiologists, triple reading by one radiologist and two radiographers may replace radiologist double-reading.

Mammography screening significantly reduces breast cancer mortality (Otto *et al*, 2003; Tabar *et al*, 2003; Berry *et al*, 2005). Compared with single-reading, double interpretation of screening mammograms improves cancer detection rates by 6–15% (Brown *et al*, 1996; Harvey *et al*, 2003; Gur *et al*, 2004; Ciatto *et al*, 2005). Double interpretation can be performed in several ways. The second radiologist may be blinded to the first interpretation (i.e., blinded double-reading) or not (i.e., independent or non-blinded double-reading). Moreover, screening programmes apply variable methods for resolving reader disagreements. A woman may be recalled if only one reader considers the mammogram abnormal, without discussion of disagreement between readers; mammograms may be interpreted in consensus, in which recall occurs only with agreement of the radiologists involved; or a decision on reader disagreement may be obtained by panel arbitration. Alternatives for double interpretation by a second radiologist include second reading by a mammography radiographer or computer-aided detection devices (Pauli *et al*, 1996; Tonita *et al*, 1999; Gilbert *et al*, 2006, 2008).

In the Netherlands, a nation-wide programme provides biennial screening mammography for women aged 50–75 years. All mammograms are double-read in a non-blinded manner. In case of a discrepant reading, the two radiologists discuss the case together to reach consensus about referral.

One previous field study found a substantial variability in mammography interpretation among radiologists, which was responsible for potential delays in breast cancer diagnosis (Gur *et al*, 2004). In many studies, mammography test sets that may not adequately represent the actual screening setting are used, and data from real-world practice are therefore sparse (Elmore *et al*, 1994; Kerlikowske *et al*, 1998; Rutter and Taplin, 2000; Esserman *et al*, 2002). We therefore prospectively determined the variability in radiologists' interpretation of screening mammograms and assessed the influence of additional reading by radiologists and radiographers on mammography screening outcome at 2-year follow-up.

## Materials and methods

### Study population

We included all 106 093 mammograms of women aged 50–75 years who underwent biennial screening mammography at two specialized, analogue screening units in the southern Netherlands between 1 January 2003 and 1 July 2006. All women had given written informed consent to use their screening and follow-up data for evaluation purposes. According to the Dutch Central Committee on Research involving Human Subjects (CCMO), approval by our local Institutional Review Board was not required.

### Screening procedure and mammogram readers

Details of the nation-wide screening programme and double-reading procedure by radiographers and radiologists have been described previously (Fracheboud *et al*, 2001; Duijm *et al*, 2004a, 2007). In brief, all 21 radiographers and 8 screening radiologists of the two units participated. Experience in screening mammography ranged from 1 to 124 months (mean, 69 months; median, 74 months) for radiographers, and from 39 to 95 months (mean, 79 months; median, 94 months) for radiologists, respectively. All radiologists read more than 6500 screening mammograms annually, and two radiologists are dedicated breast radiologists, who analyse the screening results and supervise quality assurance sessions with the other screening radiologists and radiographers.

Two radiographers double-read each mammogram at the screening site immediately after the examination was completed. At subsequent screening examinations, the radiographers could view previous screening mammograms. The radiographers decided for each mammogram whether additional work-up was required (i.e., whether the mammogram was positive). For each positive mammogram, the radiographers recorded the woman's name and date of birth, the date of screening, and the mammographic findings on a form that was developed for this study. Mammographic findings were classified according to one of five categories of abnormal findings: suspicious high density (e.g., spiculated density or density with indistinct borders), suspicious microcalcifications (e.g., pleomorphic, branching, or amorphous/indistinct microcalcifications), high density in combination with microcalcifications, architectural distortion, or breast parenchyma asymmetry. The mammograms were then double-read by two radiologists, who were blinded to the referral opinion of the radiographers. The second reader was not blinded to the opinion of the first, but aimed not to see it before making his own decision. For each discrepant reading, the second reader recorded the woman's name and date of birth, the date of screening, and the assessment of both radiologists on a form that was developed for this study. The two screening radiologists then tried to reach consensus whether referral of the woman with a discrepant reading was indicated.

### Referral

A woman was referred for additional workup (primary referral) if the mammogram was considered positive by both radiologists after initial double-reading or, in the case of a discrepant reading, if at least one radiologist considered referral necessary after consensus discussion. During monthly quality assurance sessions, mammograms that the radiographers had considered positive but that had not been referred by the radiologists were reviewed by two screening radiologists, who were now informed about the mammographic abnormalities detected by the radiographers. A woman was referred if, on review, at least one of the radiologists considered work-up to be essential (secondary referral).

### Screening follow-up

The follow-up period included the time through the next screening round, with a screening interval of approximately 2 years. For all women with a screening mammogram that was considered positive by at least one of the radiologists or radiographers, we collected data on diagnostic procedures undertaken, breast cancer diagnosis, histopathology, and TNM (tumour–node–metastases) classification (UICC, 1987) to identify screen-detected cancers. Procedures for the detection of interval cancers (interval cancers are breast cancers that are diagnosed in women after a negative screening examination) have been described previously (Duijm *et al*, 2004b). To determine whether an interval cancer could potentially have been a screen-detected cancer if all positive radiographer readings had been referred, we investigated if the mammographic abnormalities on the diagnostic films corresponded to any abnormalities registered by the radiographers at screening.

### Quality assurance

Throughout the study period, the radiologists reviewed breast cancer cases that were detected after secondary referral, as well as interval cancers. Every month, radiographers attended quality assurance sessions under the supervision of a breast radiologist. Together, they reviewed breast cancers that had been detected by radiologists only.

### Statistical analysis

Main outcome measures were referral rate, cancer detection rate (CDR, defined as the number of cancers detected per 1000 women screened), sensitivity and specificity of mammography screening, positive predictive value (PPV) of referral and tumour stages of screen detected cancers at different screening strategies. 95% confidence intervals (CIs) were calculated. Distribution of outcome variables across radiologists and reading strategies was tested with the *χ*^2^ test; a *P*-value of less than 0.05 was considered to indicate statistical significance. Pearson's correlation coefficient was calculated for correlations between reader experience, referral rate, and cancer detection rate. All data were entered into an Excel spreadsheet (Microsoft, Redmond, WA, USA) and statistical analyses were performed using SAS V9.12 (SAS Institute Inc, Cary, NC, USA).

## Results

### Single-reading by radiologists

**Figure 1:**
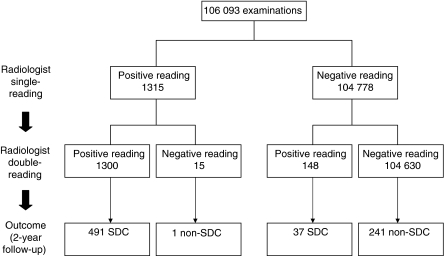
Radiologist single-reading *vs* radiologist double-reading: mammography-screening outcome at 2-year follow-up. At radiologist double-reading, a woman was referred for additional work-up if the mammogram was considered to be positive by both radiologists or, in the case of discrepant readings, if at least one radiologist considered referral necessary after consensus meeting. SDC=screen-detected cancer.

Of the 106 093 screens, 11 491 (10.8%) were initial (prevalent) screens and 94 602 (89.2%) were subsequent (incident) screens. Single-reading by radiologists would have resulted in 1315 referrals (referral rate 1.24%, 95% CI=1.17–1.31) (Figure 1). They would have detected a total of 492 cancers, corresponding to a breast cancer detection rate of 4.64 per 1000 screened women (95% CI=4.23–5.05). The sensitivity would have been 63.9% (95% CI=60.5–67.3), the specificity 99.2% (95% CI=99.2–99.3), and the PPV of referral 37.4% (95% CI=34.8–40.0) (Table 1).

Considerable individual variation existed between the radiologists regarding referral rates (range, 0.9–1.5%), breast cancer detection rates (range, 3.44–5.04 per 1000 screened women) and sensitivity (range, 51.5–75.0%; *P*=0.003). Sensitivity was significantly related to the referral rate (Pearson's correlation coefficient *r*=0.75, *P*=0.03). However, there was no correlation between radiologist experience (reading experience in number of months before the start of the study) and referral rate (Pearson's correlation coefficient *r*=0.11) or between radiologist experience and CDR (Pearson's correlation coefficient *r*=0.06).

### Radiologist double-reading

The eight radiologists formed 28 different couples for double-reading. The total number of mammograms assessed per specific couple ranged from 757 to 7484 (median: 3691). Radiologist double-reading with consensus reading would have resulted in 1448 referrals. Compared with single-reading, the referral rate would increase from 1.24 to 1.36% (95% CI=1.30–1.43, *P*=0.011; Table 2 and Figure 1). The distribution of mammographic abnormalities of the 148 additional referrals with double-reading corresponded to the distribution observed at single-reading. With double-reading and consensus discussion, 37 additional cancers were detected. One interval cancer had initially been considered positive by the first reader, but had been assessed negative at consensus reading. Double-reading increased the mean CDR from 4.64 to 4.98 (*P*=0.26), with individual radiologist values ranging from 4.44 (106/23,872; 95% CI=3.60–5.28) to 5.62 (151/26 874; 95%CI=4.73–6.51). Mean sensitivity increased from 63.9 to 68.6% (*P*=0.02), with individual radiologist values varying between 62.0% (106/171; 95% CI=54.7–69.3) and 77.0% (127/165; 95% CI=70.5–83.4). At double-reading, the two specialized breast radiologists (screener E and F) detected 67.6% (25/37) of the cancers found only by the second reader and they acquired the highest sensitivity values of 77.0% (95% CI=70.5–83.4) and 70.7% (95% CI=64.7–76.7), respectively.

Radiologist double-reading with referral of all abnormal mammograms, instead of referral following consensus in case of discrepant interpretations, would have resulted in 1473 referrals (referral rate, 1.39%, 95% CI=1.32–1.46), 530 screen detected cancers (CDR, 5.00, 95% CI=4.57–5.42), and a sensitivity of 68.8% (95% CI=65.6–72.1). This reading strategy added 25 referrals, which were considered positive only by the first reader (15 referrals) or second reader (10 referrals), to the 1448 referrals at radiologist double-reading with consensus reading.

### Referral of all positive readings at radiologist single-reading combined with radiographer double-reading

**Figure 2:**
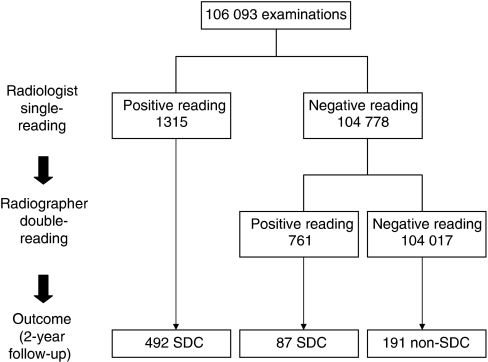
Radiologist single-reading combined with radiographer double-reading: mammography-screening outcome at 2-year follow-up. A woman was referred for additional work-up if the mammogram was considered to be positive at radiologist single-reading and/or at radiographer double-reading. SDC=screen-detected cancer.

Addition of radiographer double-reading to radiologist single-reading, in combination with referral of all mammograms that were considered abnormal by the radiologist and/or radiographers, would have resulted in a referral rate of 1.96%. (95% CI=1.87–2.04) and a CDR of 5.46 (95% CI=5.01–5.90; Table 2 and Figure 2). Compared with radiologist single-reading, additional reading by the pair of radiographers would have resulted in a significant increase of the referral rate (from 1.24 to 1.96%, *P*<0.001), the CDR (from 4.64 to 5.46, *P*=0.006), and the sensitivity (from 63.9 to 75.2%, *P*<0.001), but in a decreased PPV of referral (from 37.4 to 27.9%, *P*<0.001). The exact tumour stages of all 87 cancers, detected only by radiographers, could not be determined, as 27 of these cancers evolved as interval cancers (*n*=19) or were detected at subsequent screening (*n*=8). Given the hypothetical situation of referring all women with abnormal mammograms at triple reading, double-reading by radiologists would have led to a lower CDR of 4.98 than triple reading by one radiologist and a pair of radiographers (CDR, 5.46; *P*=0.12).

### Radiologist double-reading using secondary referral

**Figure 3:**
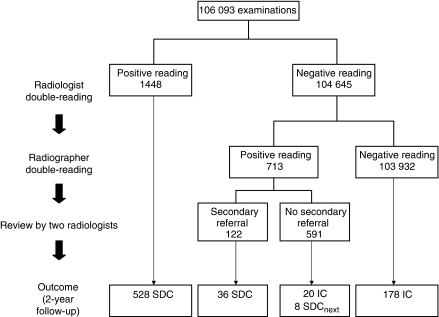
Radiologist double-reading followed by radiologist review of positive cases at radiographer double-reading: mammography-screening outcome at 2-year follow-up. SDC=screen-detected cancer; SDC_next_=cancer detected at subsequent screening; IC=interval cancer.

Radiologist double-reading, followed by review of mammograms that were considered abnormal at radiographer double-reading only, reflected the actual screening situation. Of the 713 examinations that were considered abnormal by radiographers only, 122 were referred upon review by two screening radiologists (Figure 3). Compared with cancers identified at radiologist double-reading, the 36 cancers detected after secondary referral included a higher percentage of ductal carcinomas *in situ* (22.2% (8/36) *vs* 17.0% (90/528), *P*=0.4) and a larger proportion of invasive tumours were <20 mm (T1a-c, 85.7% (24/28) *vs* 78.1% (342/438), *P*=0.3, Table 2). Replacement of screening strategy IV by strategy III (i.e., replacement of the second radiologist by a pair of radiographers, in combination with referral of all radiographer positive readings rather than review of radiographer positive readings) would have resulted in a similar cancer detection rate (5.46 *vs* 5.32, *P*=0.66) and sensitivity (75.2 *vs* 73.2%, *P*=0.22), but a lower PPV of referral (27.9 *vs* 35.9%, *P*<0.001).

### Double-reading by radiologists and radiographers with referral of all radiographer-positive readings

This screening strategy would have resulted not only in the highest referral rate (2.04%, 95% CI=1.95–2.12), but also in the highest CDR (5.58, 95% CI=5.13–6.03) and sensitivity (76.9%, 95% CI=73.9–79.9; Table 2). Compared with secondary referral of radiographer-positive readings upon review, referral of all radiographer-positive readings would have led to the detection of 28 additional cancers, including 20 interval cancers and 8 mammographic abnormalities that proved to be malignant at subsequent screening.

## Discussion

At single-reading, we observed large variations in screening outcome among the eight radiologists. The referral rates ranged from 0.9 to 1.5%, sensitivity of breast screening for cancer detection from 51.5 to 75.0% and PPV of referral from 29.5 to 45.4%. Previous studies have shown that the agreement among radiologists interpreting a test set of mammograms is relatively low (Elmore *et al*, 1994; Kerlikowske *et al*, 1998). However, data from other population-based series are very rare. The variation in CDR we observed, from 3.4 to 5.0, is comparable with the 2.6–5.4 range that was found in a US study (Gur *et al*, 2004). In line with the latter study, our results also showed that higher recall rates were significantly correlated with increased detection rates. Owing to differences between the screening programmes and study designs, further comparison between both studies is limited. The US study showed higher referral rates (range, 7.7–17.2%), PPV values were not given and sensitivity could not be calculated, as interval cancers were not included in the analysis. In a retrospective study of a random sample of screening mammograms, Elmore *et al* (2002) found a large variability range among community radiologists regarding false-positive rates, which was not eliminated after adjustment for patient, radiologist, and testing characteristics. The screening radiologists in our study had ample experience in breast screening before the start of the study, which probably explains that we found no correlation between number of months of reading experience of the radiologist and the referral rate or the CDR. Each radiologist reads a high volume of screening mammograms annually and participates in quality assurance programmes. These reading conditions might imply that their individual contributions to the skill mix has leveled off over time, but we nevertheless found large inter-observer variability in screening outcome. At double-reading, the two breast radiologists tended to have the best screening results with the highest sensitivities and the highest number of cancers detected by the second reader only, but otherwise we do not have a plausible explanation for this observation.

Our study found a 7.3% relative increase in CDR with the use of radiologist double-reading, which is comparable with those reported previously (Taplin *et al*, 2000; Harvey *et al*, 2003). The variability range in screening performance among the radiologists was reduced, but not eliminated, after double-reading. Two-thirds of the cancers, detected by the second reader only, were found by two radiologists. This observation indicates that there is room for a further increase in cancer detection by the other second readers. Beam *et al* (1996) found that specific pairs of radiologists achieve better detection rates than other pairs. We had 28 possible combinations of radiologist couples for double-reading and, taking into account the sequence of the first and second reader, even 56 couples. The numbers per specific couple varied widely and were too small to analyse Beam's belief and would be of no practical value in our screening setting; our schedules are too complex to allow specific pairs of radiologists to perform double interpretation.

Owing to a shortage of radiologists in screening programmes, such as in the United Kingdom and the United States (D'Orsi *et al*, 2005), it would be of practical value if dedicated radiographers can replace a screening radiologist as the second reader. Compared with radiologist double-reading, triple reading by one radiologist and a pair of radiographers was characterized by a 40% relative increase in a number of referrals, but an absolute referral rate of 1.96% is still low compared with other screening programmes (Smith-Bindman *et al*, 2003). The data in our study were not suited to assess the performance of individual radiologist–radiographer double-reading, as the radiographers had reported the outcome after consensus reading.

As reported previously, the highest CDR and sensitivity would have been obtained by quadruple reading by two radiologists and two radiographers, followed by referral of all radiologist and/or radiographer-positive readings (Duijm *et al*, 2007). The 2.04% referral rate at this reading strategy would still be cost effective in the Dutch screening programme (Otten *et al*, 2005; Groenewoud *et al*, 2007). There is a delicate balance between referral rate and cancer detection rate (Yankaskas *et al*, 2001). The Dutch breast screening programme is characterized by low referral rates and relatively high-interval cancer rates. In this study, we found that adding readers resulted in an increased referral rate and cancer detection rate. The current conversion of the Dutch breast screening programme from analogue to digital screening and the simultaneous introduction of screening BI-RADS categories may lower the threshold for referral (American College of Radiology, 2003; Pisano *et al*, 2005). The impact of these alterations on referral rate, detection rate and interval cancer rate is an important issue of future research.

Our study has certain limitations. First, the second readers were not fully blinded to the first reader's report, as this would be too complex in the current screening practice. The second readers aim not to see the opinion of the first reader before making their own decision about referral, but knowing the report of the first reader might have influenced their interpretation and, as a consequence, their detection rates and sensitivity concerning both single and double-reading. At present, digital mammography is introduced in the Dutch nation-wide breast cancer screening programme. The conversion from analogue to digital screening will be completed within 2 years. Non-blinded double-reading will be replaced by blinded double-reading, and the individual scores of the first and second reader will automatically be documented. The performance of each radiologist will be monitored and used for quality assurance. A second limitation of our study is the inability to assess the screening accuracy of all different pair of radiologists because the number of readings per couple sometimes was too small for interpretation. Differences between pairs may exist, however (Beam *et al*, 1996). Moreover, it is most likely that radiographer outcome parameters would have been better if only experienced radiographers were used. Although one may prefer that only experienced radiographers read mammograms, we had all radiographers participating. This approach least affected our daily screening practice, as it would be impossible to schedule specific pairs of radiographers to perform double-reading. Again, variations in the number of mammograms per specific radiographer couple precluded a proper statistical analysis of differences in screening outcome parameters among couples.

Finally, the sensitivity of breast cancer screening with 2-year follow-up will be sensitive to the number of interval cancers that developed in the second year after screening. van Dijck *et al* (1993) showed that a considerable percentage of interval cancers in a biennial screening programme appear *de novo* between two screening rounds. We related screening outcome parameters to the total breast cancer incidence after 2 years of follow-up rather than after 1 year of follow-up, as this will provide full information about the interval cancer rate and the total costs of follow-up in a biennial screening programme (Duijm *et al*, 2004b, 2008). Review of late interval cancers is part of the quality assurance and evaluation of the Dutch breast cancer screening programme.

Our prospective field study also has strengths. It is the largest study of double interpretation reported to date and is unique in assessing screening outcome from a single-reading setting to a quadruple reading setting. We were able to assess screening performance in clinical practice at different screening strategies, without having to rely on test sets. Complete follow-up data were obtained in essentially all women, allowing us to identify the false-negative examinations accurately and to calculate sensitivity of breast screening in addition to cancer detection rates.

In summary, we found large variations in individual radiologist's screening performance. Compared with single-reading, radiologists' double-reading significantly increased sensitivity. Triple reading by one radiologist and two radiographers may be an alternative to radiologist double-reading in programmes with a shortage of radiologists. Highest sensitivity was obtained by quadruple interpretation with referral of all radiologist- and radiographer-positive readings.

## Conflict of interest

The authors state no conflict of interest.

## Figures and Tables

**Table 1:** Inter-observer variability at single reading by eight radiologists (A–H): mammography screening outcome at 2-year follow-up

	**A**	**B**	**C**	**D**	**E**	**F**	**G**	**H**	**Total**
Total readings, no. (%)	23 872 (11.3)	28 244 (13.3)	26 874 (12.7)	26 754 (12.6)	26 101 (12.3)	28 317 (13.3)	25 541 (12.0)	26 483 (12.5)	212 186
*1st reading, no. (%)*	*10 178* (*42.6)*	*11 614* (*41.1)*	*12 708* (*47.3)*	*11 403* (*42.6)*	*14 493* (*55.5)*	*16 467* (*58.2)*	*15 696* (*61.5)*	*13 534* (*51.1)*	*106 093*
* 2nd reading, no. (%)*	*13 694* (*57.4)*	*16 630* (*58.9)*	*14 166* (*52.7)*	*15 351* (*57.4)*	*11 608* (*44.5)*	*11 850* (*41.8)*	*9845* (*38.5)*	*12 949* (*48.9)*	*106 093*
No. of referrals (%)	96 (0.9)	147 (1.3)	141 (1.1)	158 (1.4)	224 (1.5)	188 (1.1)	200 (1.3)	161 (1.2)	1315 (1.2)
Screen-detected cancers, no. (%)	35	57	64	57	66	80	76	57	492
CDR, per 1000 women (95% CI)	3.44 (2.30–4.58)	4.91 (3.64–6.18)	5.04 (3.81–6.27)	5.00 (3.70–6.29)	4.55 (3.46–5.65)	4.86 (3.80–5.92)	4.84 (3.76–5.93)	4.21 (3.12–5.30)	4.64 (4.23–5.05)
Non-screen-detected cancers, no.	33	32	36	19	29	41	41	47	278
Cancer prevalence, per 1000 women (95% CI)	6.68 (6.20–7.17)	7.66 (7.18–8.15)	7.87 (7.40–8.34)	6.66 (6.12–7.12)	6.55 (6.15–6.96)	7.35 (6.15–6.96)	7.45 (7.04–7.87)	7.68 (7.24–8.13)	7.26 (7.10–7.41)
Sensitivity, % (95% CI)	51.5 (39.6–63.3)	64.0 (54.1–74.0)	64.0 (54.6–73.4)	75.0 (65.3–84.7)	69.5 (60.2–78.7)	66.1 (57.7–74.5)	65.0 (56.3–73.6)	54.8 (45.2–64.4)	63.9 (60.5–67.3)
Specificity, % (95% CI)	99.4 (99.2–99.5)	99.2 (99.1–99.4)	99.4 (99.3–99.5)	99.1 (98.9–99.3)	98.9 (98.7–99.1)	99.3 (99.2–99.5)	99.2 (99.1–99.3)	99.2 (99.1–99.4)	99.2 (99.1–99.3)
PPV, % (95% CI)	36.5 (26.8–46.1)	3 8.8 (30.9–46.7)	45.4 (37.2–53.6)	36.1 (28.6–43.6)	29.5 (23.5–35.4)	42.6 (35.5–49.6)	38.0 (31.3–44.7)	35.4 (28.0–42.8)	37.4 (34.8–40.0)

CDR=cancer detection rate; CI=confidence interval; PPV=positive predictive value of referral.

**Table 2:** Breast cancers and tumour characteristics at different reading strategies

**Reading strategy**	**Single radiologist reading**	**Radiologist double-reading**	**Single radiologist reading and radiographer double-reading with referral of all positive readings**	**Radiologist double-reading followed by radiologist review of positive cases at radiographer double-reading**	**Double-reading by radiologists and radiographers with referral of all positive readings**
Referral rate, % (95% CI)	1.24 (1.17–1.31)	1.36 (1.30–1.43)	1.96 (1.87–2.04)	1.48 (1.41–1.55)	2.04 (1.95–2.12)
Mammographic abnormality, no. (%)	1315	1448	2076	1570	2161
Density	877 (66.7)	967 (66.8)	1386 (66.8)	1041 (66.3)	1448 (67.0)
Microcalcifications	257 (19.5)	289 (20.0)	447 (21.5)	331 (21.1)	464 (21.5)
Density with microcalcifications	106 (8.1)	114 (7.9)	140 (6.7)	116 (7.4)	145 (6.7)
Architectural distortion	44 (3.3)	47 (3.2)	62 (3.0)	51 (3.2)	64 (3.0)
Breast parenchyma asymmetry	31 (2.4)	31 (2.1)	41 (2.0)	31 (2.0)	40 (1.9)
Breast cancers, no.	492	528	579	564	592
CDR, per 1,000 women (95% CI)	4.64 (4.23–5.05)	4.98 (4.55–5.40)	5.46 (5.01–5.90)	5.32 (4.88–5.75)	5.58 (5.13–6.03)
Sensitivity, % (95% CI)	63.9 (60.5–67.3)	68.6 (65.3–71.9)	75.2 (72.1–78.2)	73.2 (70.1–76.4)	76.9 (73.9–79.9)
Specificity, % (95% CI)	99.2 (99.2–99.3)	99.1 (99.1–99.2)	98.6 (98.5–98.7)	99.0 (99.0–99.1)	98.5 (98.4–98.6)
PPV of referral, % (95% CI)	37.4 (34.8–40.0)	36.5 (34.0–38.9)	27.9 (26.0–29.8)	35.9 (33.6–38.3)	27.4 (25.5–29.3)
					
*Type of breast cancer, no. (%)*					
DCIS	80 (16.3)	90 (17.0)	NA	98 (17.4)	NA
Invasive	412 (83.7)	438 (83.0)	NA	466 (82.6)	NA
T1a–c	321 (77.9)	342 (78.1)	NA	366 (78.5)	NA
T2	88 (21.4)	93 (21.2)	NA	96 (20.6)	NA
Unknown	3 (0.7)	3 (0.7)	NA	4 (0.9)	NA

CI=confidence interval; CDR=cancer detection rate; DCIS=ductal carcinoma *in situ*; PPV=positive predictive value.

NA: several cancers were diagnosed as interval cancers or detected at subsequent screening. Consequently, exact tumour stages of these cancers at the time of the index screening examination are not available.
